# Overproduction of Native and Click-able Colanic Acid Slime from Engineered *Escherichia coli*

**DOI:** 10.1021/jacsau.2c00583

**Published:** 2023-02-03

**Authors:** Joanna C. Sadler, Richard C. Brewster, Annemette Kjeldsen, Alba F. González, Jessica S. Nirkko, Simon Varzandeh, Stephen Wallace

**Affiliations:** Institute of Quantitative Biology, Biochemistry and Biotechnology, School of Biological Sciences, University of Edinburgh, King’s Buildings, Alexander Crum Brown Road, Edinburgh EH9 3FF, U.K.

**Keywords:** biotechnology, synthetic biology, exopolysaccharide, click chemistry, biopolymer engineering

## Abstract

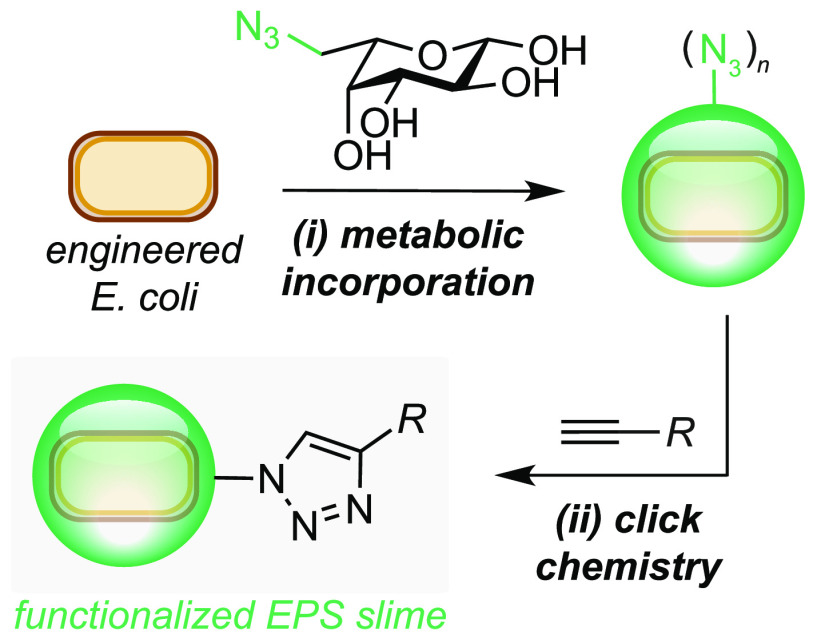

The fundamental biology and application of bacterial exopolysaccharides is gaining increasing attention. However, current synthetic biology efforts to produce the major component of *Escherichia sp.* slime, colanic acid, and functional derivatives thereof have been limited. Herein, we report the overproduction of colanic acid (up to 1.32 g/L) from d-glucose in an engineered strain of *Escherichia coli* JM109. Furthermore, we report that chemically synthesized l-fucose analogues containing an azide motif can be metabolically incorporated into the slime layer via a heterologous fucose salvage pathway from *Bacteroides sp.* and used in a click reaction to attach an organic cargo to the cell surface. This molecular-engineered biopolymer has potential as a new tool for use in chemical, biological, and materials research.

Exopolysaccharide slimes (EPS) are produced by many bacteria in response to external environmental stress. These polymeric carbohydrates are rapidly synthesized in the cell interior and exported to the cell surface to encapsulate the host in a protective slime-like layer. Colanic acid (CA) is the major exopolysaccharide produced by *Escherichia sp.* and has been shown to protect cells from toxic metal ions,^1−3^ host organisms from pathogenic microorganisms,^4^ and, most recently, to delay the effect of aging in a microbe-associated host.^4,5^ Colanic acid therefore has the potential to be used for a range of applications, including biomaterial design, drug delivery, and cosmetics research. In biocompatible chemistry, slime production has recently been shown to mitigate the damaging effects of hydrophobic metabolites and membrane-active surfactants. Nanomicelles containing a transition-metal catalyst were shown to induce CA slime formation in *E. coli* NST74, and this increased cell viability.^6^ Interestingly, slime production had no negative effect on (i) metabolite flux through an engineered styrene production pathway or (ii) the efficiency of an Fe-catalyzed cyclopropanation reaction at the cell surface. Slime formation could therefore be a viable approach to mitigate the otherwise toxic effects of engineered metabolites and/or nonenzymatic reactions to bacterial cells. Thus, the bioproduction of CA and its derivatives from renewable resources via synthetic biology is an important challenge for industrial biotechnology.

The chemical structure of colanic acid is heterogeneous, yet it contains a repeating unit of d-glucose (Glu), l-fucose (Fuc), d-galactose (Gal), and d-glucuronic acid (GlcA), which together make the slime negatively charged (Figure 1A).^7−9^ The biosynthesis of CA is encoded by the 21-gene, 24 kb *wca* operon (formerly named *cps*) that is ubiquitous to *Escherichia sp.* (Figure 1B).^8^ Transcription is initiated via a JUMPStart-RfaH antitermination mechanism^10,11^ and is positively regulated by the transcriptional activators RcsA and RcsB.^12,13^ Interestingly, plasmid-based overexpression of RcsA has been demonstrated as a viable engineering strategy to produce increased quantitates of CA from d-glucose (up to 350 mg/L).^1^

**Figure 1:**
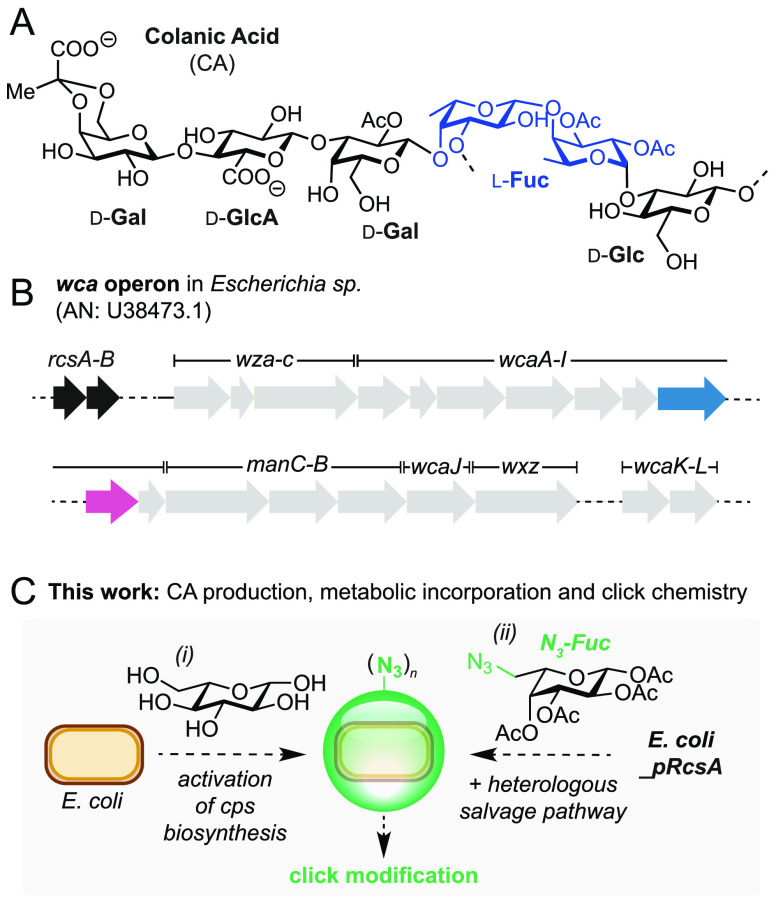
(A) The structure of the major repeating unit in CA. l-Fucose units are highlighted in blue. (B) The *wca* operon in *Escherichia sp*. The positive regulators of CA biosynthesis, RcsA/B, are highlighted in black; *gmd* and *fcl* are highlighted in blue and pink, respectively. (C) Optimization of CA production and incorporation of non-natural sugars. AN = accession number.

Inspired by these studies and recent interest in colanic acid from the biological community, we set out to increase the bioproduction of CA and develop a method to modify its structure via the metabolic incorporation of an unnatural sugar. This is motivated by our interest in localizing nonenzymatic catalysts to the cell surface for use in new biocompatible reactions. Herein we report the optimization and subsequent high-level production of CA from d-glucose (1.3 g/L) in *E. coli* JM109Δ*waaF*_pRcsA under optimized fermentation conditions. Finally, we demonstrate the metabolic incorporation of the azide-containing unnatural sugar, Fuc-N_3_, into CA using a fucose salvage pathway from *Bacteroides sp.*(14) and conduct preliminary investigations into the use of chemically engineered slime to localize cargo to the bacterial outer membrane via fluorescence detection (Figure 1C).

We began by comparing RcsA-modified *E. coli* to other Gram-negative microorganisms that are known to produce acidic slime. We chose the organisms *Azotobacter vinelandii*, *Zooglea ramigera*, and *Sinorrhizobium meliloti*, which are known to produce alginate, zooglan, and succinoglycan polysaccharides, respectively.^15−17^*E. coli* BL21(DE3) and TOP10 cells transformed with a plasmid encoding RcsA (pRcsA) were used alongside an unmodified BL21(DE3) control. Slime formation was assayed by observing colony phenotype on agar plates supplemented with isopropyl β-d-1-thiogalactopyranoside (IPTG) to induce expression of RcsA (Figure 2A). Out of the *E. coli* strains tested, JM109_pRcsA was the only strain to produce a lustrous, shiny phenotype, which was indicative of CA overproduction. Conversely, BL21(DE3)_pRcsA and TOP10_pRcsA phenotypes were comparable to that of the JM109 empty vector negative control (JM109_pEdinbrick). This result was confirmed by measuring total carbohydrate and CA production in liquid cultures of BL21(DE3), TOP10, and JM109 transformed with pEdinbrick (empty vector control) or pRcsA (Figure 2B). Strain JM109_pRcsA gave more than five-fold higher total carbohydrate titers and was the only strain to generate detectable levels of CA. This result was in agreement with the hypothesis that JM109 is deficient in Lon protease, which degrades RcsA^12,18^ and, unlike other common laboratory *E. coli* strains such as BL21(DE3) and TOP10, does not carry *gal* mutations resulting in lower levels of CA building block UDP-d-galactose.^19,20^

**Figure 2:**
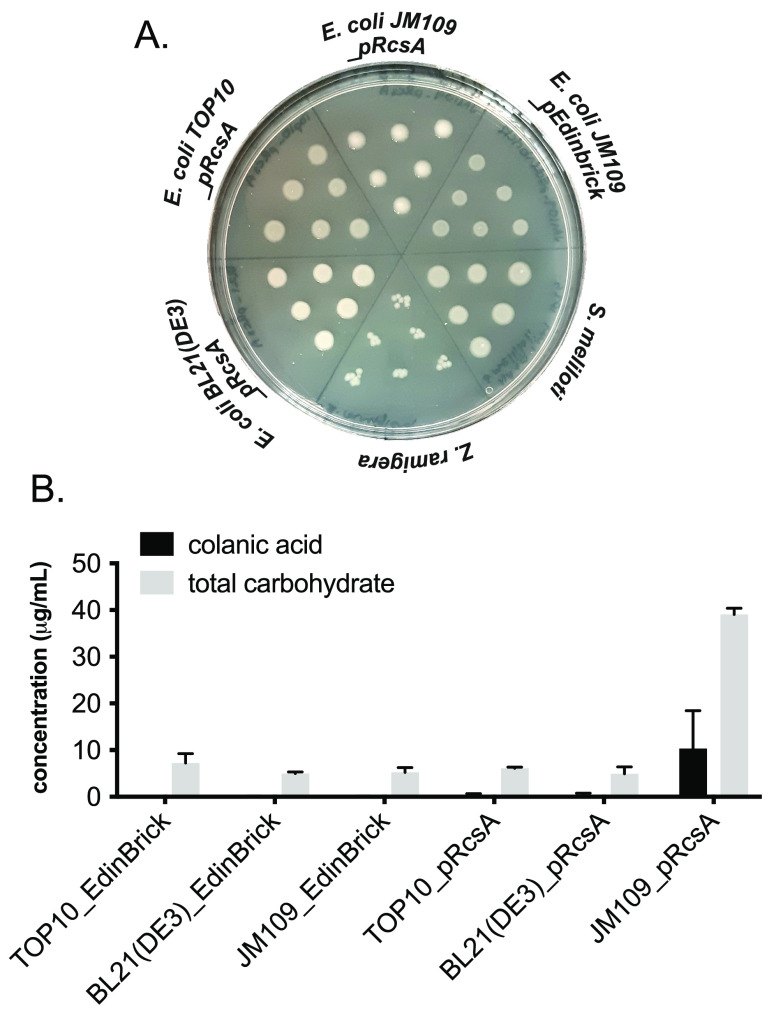
(A) Solid-phase screening for slime production on agar plates. Slime formation results in a lustrous, shiny colony phenotype. (B) Liquid-phase screening for total carbohydrate and CA production by modified *E*. *coli* strains. CA was quantified by measuring fucose concentration in hydrolyzed samples by derivatization of extracted EPS with Cys·HCl and measuring the difference in absorbance at 427 and 396 nm. Total carbohydrate was quantified via the anthrone assay and measuring the absorbance at 620 nm. Data from quantitative experiments are presented as averages of three independent experiments to one standard deviation.

To maximize CA production by JM109_pRcsA, a series of optimization experiments was carried out. First, a media screen identified M9 and MDM, both minimal media, to provide the highest CA yields. Due to very slow growth rates and poor reproducibility in MDM, M9 was selected for all further investigations (Figure S6). Glucose concentration could be decreased from 5% to 0.5% w/v without a significant effect on CA yield (Figure S7). The nitrogen source also had a profound effect on CA yields, with proline proving superior to both ammonium chloride and ammonium sulfate (Figures S8 and S9). The effect of the incubation temperature both in the presence and absence of trace amounts of Cu^2+^ and Fe^2+^ was also studied. A lower postinduction incubation temperature of 19 °C compared to 37 °C was found to increase CA yields to approximately 80% of the total carbohydrate content of the EPS (Figure 3A). The addition of Fe^2+^ and Cu^2+^ alone did not have a significant effect on CA yields at any temperature; however, they did increase growth rates of cultures. CA production over time was also studied. Both total carbohydrate and CA yields increased throughout the exponential growth phase, after which there was a small decrease in CA yields, despite a continued increase in total carbohydrate content (Figure 3B). Taking all of the optimization experiments together, the optimal conditions for CA production were concluded to be 90 h of incubation at 19 °C in M9 minimal media containing trace levels of Fe^2+^ and Cu^2+^, proline as the nitrogen source, and 0.5% w/v glucose, yielding 419 mg/L CA (vs 77 mg/L in the *E. coli* JM109_pEdinbrick control). As a final optimization to further improve CA levels we knocked out the *waaF* gene using a λ-Red recombinase. *WaaF* (also annotated as RfaF) is involved in carbohydrate tailoring during lipopolysaccharide (LPS) biosynthesis in Gram-negative bacteria, and *ΔwaaF* strains have been shown to produce increased levels of EPS.^19^ Indeed, this increased CA titers in the host strain to 719 mg/L, which increased further to 1.32 g/L when combined with *RcsA* overexpression (Figure 3C).

**Figure 3:**
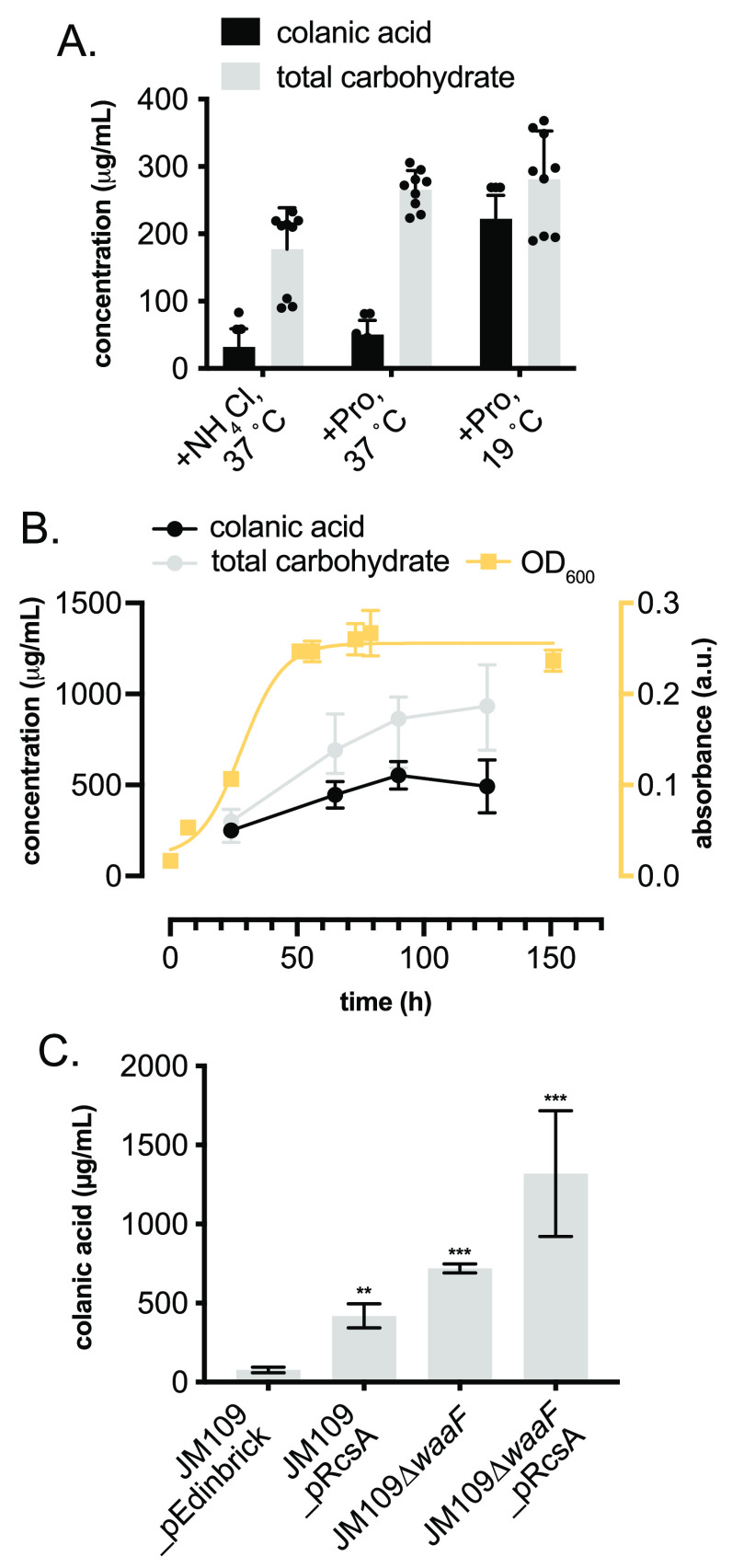
(A) Temperature and N-source screen to increase total carbohydrate and CA production in *E*. *coli* JM109_pRcsA. (B) CA production over time related to culture growth phase and total carbohydrate production. (C) Strain optimization to enhance CA production. CA was quantified by measuring fucose concentration in hydrolyzed samples by derivatization of extracted EPS with Cys·HCl and measuring the difference in absorbance at 427 and 396 nm. Total carbohydrate was quantified via the anthrone assay and measuring the absorbance at 620 nm. Data from quantitative experiments are presented as averages of three independent experiments to one standard deviation. *P < 0.05, **P < 0.005, ***P < 0.0005..

With conditions for increased levels of CA containing slime production in hand, we set out to functionalize the slime by metabolic incorporation of non-natural sugars. While this is well-established in mammalian cell lines,^20^ production of non-natural EPS analogues by *E. coli* is less established. A notable development in this field was the remodeling of bacterial polysaccharides by use of an exogenous sugar nucleotide salvage pathway.^14^ In this work, the de novo guanosine diphosphate (GDP) fucose pathway comprising GDP-mannose dehydratase (GMD) and GDP-fucose synthetase (Fcl) was deactivated in a Δ*gmd-fcl* strain of *E. coli* (Figure 1B) and a salvage pathway activated by heterologous expression of the *fkp* gene, which encodes a bifunctional protein from *Bacteroides fragilis*(21) with (i) fucokinase and (ii) GDP-fucose pyrophosphorylase activity. Following a similar strategy, we envisioned that unnatural azide-containing sugars could be introduced into bacterial slime via metabolic incorporation and that the resulting EPS could be functionalized using click chemistry. To this end, we prepared the fucose azide analogue Fuc-N_3_ in five steps from l-galactose via nucleophilic addition of sodium azide to the triflate generated from the primary alcohol of the corresponding *bis*-isopropylidene acetal, followed by deprotection in 30% overall yield (Figure 4).^22^ To determine whether the salvage pathway was required for the incorporation of Fuc-N_3_, we quantified fucose levels from Δ*gmd-fcl* strains grown in the presence and absence of fucose and Fuc-N_3_. The knockout strain was prepared using a λ-Red recombinase and then transformed with pRcsA and a plasmid encoding the *fkp* gene from *Bacillus subtilis* (pFkp; Table S2). No fucose incorporation was observed in Δ*gmd-fcl* cells expressing *rcsA* when grown in the presence of glucose, glucose and fucose, or glucose and Fuc-N_3_, indicating the need for an alternative supply of GDP-fucose for CA biosynthesis in this strain. However, *Δgmd-fcl* cells cotransformed with pRcsA and pFkp produced more than 100 mg/L CA when grown in the presence of Fuc, but no CA was detected in the presence of glucose and Fuc-N_3_. This indicated that the salvage pathway from *Bacteroides sp.* was either unable to accept Fuc-N_3_ or was being poorly expressed in *E. coli* JM109. Analysis by sodium dodecyl sulfate polyacrylamide gel electrophoresis (SDS-PAGE) indicated low levels of Fkp in cells, and therefore we carried out a series of optimization experiments to increase protein expression. A screen of pre- and post-induction conditions revealed that Fkp levels could be significantly increased if cells were grown at 37 °C to an exponential phase and then cooled to room temperature after induction with IPTG (Figure S10). Pleasingly, growth of JM109Δ*gmd-fcl* cells expressing pRcsA under these optimized conditions and in the presence of glucose and Fuc-N_3_ resulted in the production of full-length colanic acid EPS (Figure 5B). To confirm the incorporation of Fuc-N_3_ into the EPS we conducted a labeling experiment using a copper-catalyzed azide–alkyne cycloaddition (CuAAc) reaction (Figure 5A). The water-soluble ligand THPTA (*tris*((1-hydroxy-propyl-*1H*-1,2,3-triazol-4-yl)methyl)amine) was chosen instead of TBTA (*tris*(benzyltriazolylmethyl)amine) to increase penetration of the EPS, stabilize Cu(I), and disfavor oxidative side-reactions. The fluorescent alkyne-containing dye 5-fluorescein-alkyne (5-FAM-alkyne) was chosen due to its known reactivity in the CuAAc reaction under aqueous conditions and visible excitation and emissions wavelengths at 485 and 520 nm, respectively. Therefore, azide EPS from JM109Δ*gmd-fcl*_pRcsA_pFkp cells was incubated with 5-FAM-alkyne in the presence of Cu(II)THPTA for 16 h, dialyzed, and then analyzed by fluorescence spectroscopy. Pleasingly, fluorescent triazole-linked EPS was only detected in samples expressing *fkp* that had been incubated with Fuc-N_3_ and Cu(II) catalyst, confirming both the metabolic incorporation of Fuc-N_3_ through *wca* biosynthesis and the functional activity of the resulting EPS slime toward covalent attachment of an external small molecule (Figure 5C). Finally, outer membrane colocalization of 5-FAM-labeled EPS on engineered *E. coli* JM109 cells was confirmed by fluorescence microscopy (Figures 5D, S11, and S12). Given the importance of EPS layers in bacterial biofilms, their pathogenicity in humans, and the growing interest in colanic acid as a biocompatible polymeric material, this technology paves the way for the chemical design of new functional biomaterials for a variety of applications through metabolic incorporation and *in vivo* click chemistry in engineered bacteria. Colanic acid M-antigen is also tightly associated with the outer membrane after export by the Wzx flippase, and therefore click-modified exopolysaccharide slimes could also provide a targeting methodology for the selective delivery of small molecules to bacterial cells in multicellular environments.

**Figure 4:**
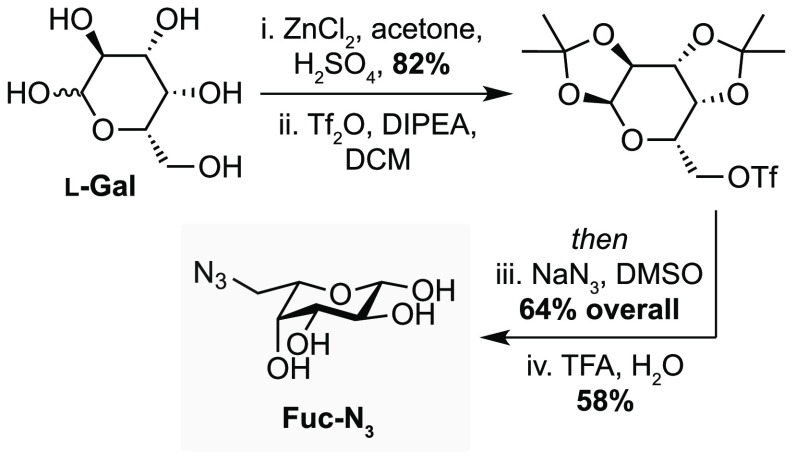
Synthesis of azide-containing fucose analogue Fuc-N_3_.

**Figure 5:**
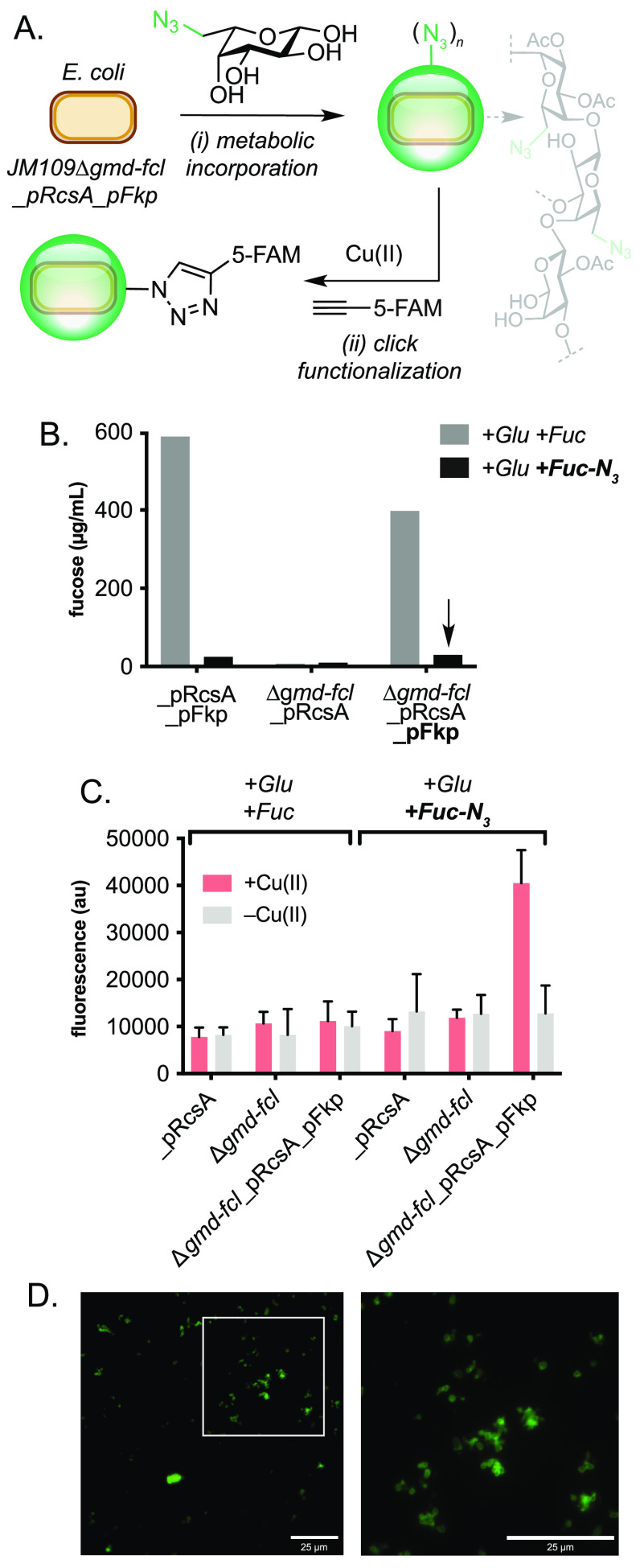
Click functionalization of azide-modified slime. (A) Metabolic incorporation of Fuc-N_3_ in engineered *E. coli* JM109 and CuAAc labeling of a fluorescent dye. (B) EPS production in engineered strains containing native and heterologous fucose salvage pathways when cultured in the presence of Fuc and Fuc-N_3_. (C) Fluorescent read-out from click-modified EPS generated by strains engineered to metabolically incorporate Fuc-N_3_. (D) Fluorescence microcopy images of FAM-labeled EPS on the surface of engineered *E. coli* JM109. Scale bars = 25 μM. Fucose was quantified by acid hydrolysis and addition of l-Cys, followed by spectrophotometric detection at 427 and 396 nm. Click reactions were performed using CuSO_4_ (100 μM), THPTA (500 μM), 5-FAM-alkyne (50 μM), and sodium ascorbate (5 mM) in potassium phosphate buffer (pH7, 100 mM), at 30 °C and 1000 rpm. Fluorescence was measured at λ_ex_ = 485 nm and λ_em_ = 520 nm. THPTA = *tris*((1-hydroxy-propyl-1*H*-1,2,3-triazol-4-yl)methyl)amine. FAM = carboxyfluorescein. Data is presented as an average of three independent experiments to one standard deviation.

To conclude, this study reports the overproduction and chemo-enzymatic synthesis of modified bacterial exopolysaccharide slime from engineered *Escherichia coli*. High-level production of colanic acid EPS was achieved through deregulation of the *wca* operon and redirection of carbohydrate away from LPS biosynthesis in *E. coli* JM109 and optimization of growth conditions (temperature and N-source). Unnatural azide-containing fucose (Fuc-N_3_) was synthesized and metabolically incorporated into the slime layer by deletion of native fucose pathways (Δ*gmd-fcl*) combined with heterologous expression of a fucose salvage pathway from *Bacteroides sp*. When combined in a JM109Δ*gmd-fcl*_pRcsA_pFkp engineered strain, azide-modified EPS slime could be produced at room temperature and modified via a Cu-catalyzed azide–alkyne click reaction to produce a fluorescent product that colocalizes to the cell surface. This is the first production of such quantities of functionalized EPS slime from a metabolically engineered bacterium. Further metabolic engineering of this system to improve the production of both native and azide-modified EPS from other carbon feedstocks and d-Glu containing waste streams is currently underway in our laboratory. Overall, we anticipate broad utility of these bioproduced and chemically functionalized microbial exopolysaccharides across the chemical and biological sciences.

## Methods

### Colanic Acid Production under Optimized Conditions

Lysogeny broth (LB) containing ampicillin (100 μg/mL) was inoculated with a single colony from freshly streaked plates of JM109_pRcsA or JM109_pEdinbrick and incubated overnight at 37 °C with shaking (220 rpm). The next day, M9 EPS media containing ampicillin (100 μg/mL) was inoculated with 2% v/v overnight culture and incubated at 19 °C for 24 h. IPTG was added (100 μM final concentration), and cultures were incubated at 19 °C for a further 72–96 h.

### Colanic Acid Extraction

Bacterial cultures were pelleted by centrifugation (70 000*g*, 4 °C, 30 min), and the resulting supernatant was transferred to a clean Falcon tube. Three volumes of acetone were added to 1 volume of supernatant and incubated at 4 °C overnight. The resulting suspension was centrifuged (13 000 rpm, 4 °C, 30 min) before the supernatant was carefully removed to leave the pellet undisturbed. The pellet was dissolved in Milli-Q water (5–10 mL per 10 mL of culture volume), and the resulting solution was dialyzed against Milli-Q water for 16 h at room temperature, using a 3.5 kDa molecular weight cutoff dialysis membrane. The dialysis cassette was transferred into fresh Milli-Q water and dialyzed for a further 4 h at room temperature. The resulting EPS solution was analyzed directly for colanic acid or freeze-dried to provide solid EPS samples.

### Colanic Acid Quantification

Quantification of colanic acid was carried out based on the protocol, reported by Obadia and co-workers,^23^ that measures fucose concentration, a constituent of EPS. Purified EPS samples were diluted in Milli-Q H_2_O depending on their predicted concentration to fit within the standard curve range. Triplicate runs were carried out by mixing 444 μL of the diluted samples with 2 mL of H_2_SO_4_/H_2_O (6:1 v/v) in a glass tube. The mixture was then heated to 95 °C for 30 min and then cooled to room temperature. For each sample, 20 μL each of (i) a freshly prepared cysteine hydrochloride (Cys·HCl, 3% (w/v)) solution and (ii) Milli-Q water was added to a polystyrene cuvette. One milliliter of the EPS sample was added, and the absorbance of each cuvette was measured at both 396 and 427 nm. Absorbance measurements without Cys·HCl were subtracted from those with Cys·HCl to provide corrected *A*_396_ and *A*_427_ values and then correlated to fucose concentration by using a fucose standard curve ranging from 5 to 100 μg/mL (Figure S3). This protocol was also miniaturized for 96-well plate format, as described in the Supporting Information.

### Production of Azide-Labeled Colanic Acid

LB (10 mL) containing ampicillin (100 μg/mL) and kanamycin (50 μg/mL) was inoculated with a single colony from freshly streaked plates of JM109(DE3)Δ*gmd-fcl*_pRcsA_pFkp and incubated overnight at 37 °C with shaking (220 rpm). The next day, M9 EPS media containing ampicillin (100 μg/mL), kanamycin (50 μg/mL), and 0.1% w/v Fuc-N_3_ was inoculated with 2% v/v overnight culture and incubated at 37 °C until an OD_600_ of 0.3–0.4 was reached. IPTG was added (500 μM final concentration), and cultures were incubated at 19 °C for a further 72 h.

### Fluorescent Labeling of Azide-Labeled Colanic Acid

Cultures containing azide-labeled colanic acid were diluted to OD_600_ = 1 in sterile phosphate-buffered saline (PBS) and dialyzed against PBS for 24 h using a 3.5 kDa molecular weight cutoff membrane. 250 μL of the dialyzed suspension was added to an Eppendorf tube followed by a premixed solution of CuSO_4_ (100 μM final concentration) and *tris*(benzyltriazolylmethyl)amine (THPTA, 500 μM final concentration), 5-FAM alkyne (50 μM final concentration), sodium ascorbate (5 mM final concentration), and potassium phosphate buffer (pH 7, 100 mM final concentration and a final reaction volume of 500 μL). Reactions were incubated in a thermoshaker at 30 °C (1000 rpm) for 16 h before dialyzing a 100 μL aliquot against Milli-Q water for 7 days, changing the dialysis water every 24 h. Samples were diluted with Milli-Q water to a constant volume of 200 μL, and fluorescence was determined by spectrophotometry (λ_ex_ = 485 nm, λ_em_ = 520 nm).

## Supporting Information Available

Supplementary data, general materials and methods, molecular biology procedures and synthesis information are available, including DNA sequence information and ^1^H/^13^ NMR characterization data (PDF)
