# Supplementary material for: Overproduction of Native and Click-able Colanic Acid Slime from Engineered Escherichia coli
Source: JACS Au. 2023 Feb 3;3(2):378–83. doi: 10.1021/jacsau.2c00583 (PMC9976346; doi:10.1021/jacsau.2c00583)
Supplement: Supplementary file 1 — au2c00583_si_001.pdf [file au2c00583_si_001.pdf]

Supplementary Information

Overproduction of Native and Click-able Colanic Acid Slime from Engineered *Escherichia coli*

Joanna C. Sadler, Richard C. Brewster, Annemette Kjeldsen, Alba F González, Jessica S. Nirkko, Simon Varzandeh and Stephen Wallace*

Institute of Quantitative Biology, Biochemistry and Biotechnology, School of Biological Sciences, University of Edinburgh, Roger Land Building, Alexander Crum Brown Road, King's Buildings, Edinburgh, EH9 3FF

**Correspondence to:* stephen.wallace@ed.ac.uk

Contents

1 General materials and methods 3

1.1 NMR spectroscopy 3

1.2 Media recipes 3

2 Molecular biology 4

2.1 General procedure for the preparation of knockout strains 5

2.2 Strains and plasmids 6

3 Supplementary quantification methods 8

3.1 Colanic acid quantification using plate reader 8

3.2 Quantitative analysis of total carbohydrate content 9

4 EPS production supplementary methods 9

4.1 Optimization of colanic acid production 9

4.2 Procedure for preparation of fluorescence microscopy samples 10

5 Synthesis 11

6 Supplementary data 12

7 References 17

1 General materials and methods

Unless otherwise stated, starting materials and reagents were obtained from commercial suppliers and were used without further purification. All water used experimentally was purified with a Suez Select purification system (18 MΩ.cm, 0.2 µM filter).

1.1 NMR spectroscopy

Proton nuclear magnetic resonance spectra (^{1}H NMR) were recorded using a Bruker AVA400, AVA500, Pro500 or AVA600 NMR spectrometer at the specified frequency at 298 K. Proton chemical shifts are expressed in parts per million (ppm, δ scale) and are referenced to residual protium in the NMR solvent (DMSO-d_6 = 2.50 ppm). Carbon nuclear magnetic resonance spectra (^{13}C NMR) were recorded using a Bruker AVA400, AVA500, Pro500 or AVA600 NMR spectrometer at the specified frequency at 298 K. Chemical shifts are quoted in parts per million (ppm, δ scale) and are referenced to the carbon resonances of the NMR solvent (DMSO-d_6 = 39.5 ppm). Coupling constants, *J*, are measured to the nearest 0.1 Hz and are presented as observed. Data is represented as: chemical shift, integration, multiplicity (s = singlet, d = doublet, t = triplet, q = quartet, dd = doublet of doublets, m = multiplet and/or multiple resonances), coupling constant (*J*) in Hertz. All NMR spectroscopy solvents were purchased from commercial suppliers.

1.2 Media recipes

Lysogeny Broth (LB) Medium

Bacto-tryptone (10 g/L), yeast extract (5 g/L) and NaCl (10 g/L) were dissolved in Milli-Q water. LB was autoclaved at 121 ˚C for 20 minutes, cooled and stored at room temperature. LB agar was prepared using the same recipe but with the addition of agar (15 g/L).

2XTY (Tryptone yeast) medium

Bacto-tryptone (16 g/L), yeast extract (10 g/L) and NaCl (5 g/L) were dissolved in Milli-Q water. 2XTY was autoclaved at 121 ˚C for 20 minutes, cooled and stored at room temperature.

M9 minimal medium

A 5X M9 stock solution was prepared as follows: $Na_2HPO_4{\cdot}12H_2O$ (85.5 g/L), KH_2PO_4 (15 g/L), NaCl (2.5 g/L), NH_4Cl (5 g/L) and L-proline (27 g/L) were dissolved in Milli-Q water and autoclaved at 121 ˚C for 20 minutes. To prepare the final solution, filter sterilized $MgSO_4$ (2 mL, 1 M stock solution), $CaCl_2$ (2 mL, 50 mM stock solution), thiamine hydrochloride (0.8 mL, 50 mg/mL stock solution), $FeSO_4$ (0.1 mL, 10 mg/mL stock solution), $CuSO_4$ (0.1 mL, 10 mg/mL stock solution) and autoclaved glucose (25 mL, 20% w/v stock solution) were added to 200 mL 5X M9 stock solution and the final volume was adjusted to 1 L with sterile Milli-Q water.

M9 CA minimal medium

M9 minimal medium was prepared as described above, with the addition of 25 g/L casamino acids being added to the 5X M9 stock solution, for a final concentration of 5 g/L casamino acids.

MDM medium

A 4X MDM stock solution was prepared as follows: $Na_2HPO_4 \cdot 7H_2O$ (64 g/L), KH_2PO_4 (15 g/L), NaCl (2.5 g/L) and NH_4Cl (5 g/L) was dissolved in Milli-Q H_2O and autoclaved at 121 °C for 20 minutes then cooled to room temperature. The mixture was diluted to 1X and filter sterilized thiamine hydrochloride (0.25 mM final concentration) and sterile glucose solution (1.25% w/v final concentration) were added. The mixture was supplemented with filter sterilized $MgSO_4$ (2 mL, 1 M stock solution), $CaCl_2$ (2 mL, 50 mM stock solution), thiamine hydrochloride (0.8 mL, 50 mg/mL stock solution), $FeSO_4$ (0.1 mL, 10 mg/mL stock solution) and $CuSO_4$ (0.1 mL, 1 mg/mL stock solution) and made up to 1 L final volume with Milli-Q water to make 1X MDM medium.

YESCA medium

0.5 g/L yeast extract and 5 g/L casamino acids were dissolved in Milli-Q water and autoclaved at 121 °C for 20 minutes then cooled to room temperature.

2 Molecular biology

The *fkp* gene (Table 1) was codon-optimized for *E. coli* BL21(DE3) and synthesized using GeneArt™ (Thermo Scientific). Oligonucleotide primers were synthesized by Integrated DNA Technologies. Recombinant plasmid DNA was purified with a Miniprep Kit (Qiagen). *E. coli* strain JM109_pRcsA was kindly provided by the laboratory of Prof. French (University of Edinburgh). pRSFDuet-1 was purchased from Novagen. All restriction enzymes were purchased from Thermo Fisher as FastDigest™ enzymes. All restriction enzyme digests were carried out at 37 °C using FastDigest™ Green buffer. All plasmids were sequenced by Sanger sequencing at Edinburgh Genomics (Edinburgh, UK).

OneTaq 2X premix (New England Biolabs) was used for all colony PCR reactions, which contained 0.5 µM forward and reverse primers and water to a final volume of 12.5 µL. Phusion High-Fidelity DNA Polymerase (New England Biolabs) was used for all other PCR reactions, which contained 0.5 µM forward and reverse primers, 200 µM each dNTP, 10-20 ng template DNA and 1X HF buffer. T4 DNA ligase (Thermo Scientific) was used for all ligation reactions.

Colony PCR reactions were performed using the following conditions: initial denaturation (95 °C, 10 minutes), 35 thermal cycles (20 seconds denaturation at 95 °C, annealing at 50-65 °C for 30 seconds, and extension at 68 °C for 60 s/kb), and final extension (68 °C for 10 minutes). All standard Phusion PCR reactions were performed using the following conditions: initial denaturation (98 °C, 30 seconds), 30 thermal cycles (15 seconds denaturation at 98 °C, annealing at 50-72 °C for 20 seconds, and extension at 72 °C for 30 s/kb), and final extension (72 °C for 7 minutes). For agarose gel

electrophoresis, agarose (1% w/v) TAE gels containing a 1 kB GeneRuler ladder (Thermo Scientific) were run at 100 V for 40 minutes and visualized using SYBR SafeTM. For SDS-PAGE, 12-well 12% acrylamide Bis-Tris NuPAGE gels (Thermo Scientific) containing an unstained Precision Plus standard ladder (BioRad) were used to analyze samples. Gels were run in 1X MES buffer (Novagen) at 50 V for 30 minutes followed by 150 V for 2 hours.

2.1 General procedure for the preparation of knockout strains

E. coli JM109(DE3)*Δgmd-fcl* and JM109*ΔwaaF* were generated via the reported protocol by Court *et. al*[1] with the following modifications: (i) the linear CamR cassette was purified via gel extraction and the coding DNA sequence was amplified a second time via PCR, (ii) cells were washed with glycerol (10% v/v aqueous solution) when preparing electrocompetent cells and were electroporated at 2.5 kV (200 Ω, 25 µFd), (iii) cultures were grown at 37 °C for 5 h following electroporation. The pSIM27 plasmid was used for recombineering and was obtained from the Court Lab (National Institute of Health, MD, USA). The chloramphenicol resistance cassette was constructed using the primers detailed in Table S1 using an annealing temperature of 60 ˚C under standard PCR conditions. Knockout colonies were confirmed via colony PCR using primers *gmd-fcl*_A-D or *waaF*_A-D (Table S1) as appropriate using an annealing temperature of 60 ˚C under standard colony PCR conditions.

Table S1. *Primers used for the preparation of JM109(DE3)Δgmd-fcl and JM109ΔwaaF knockout strains.*

Primer number	Primer name	Primer sequence (5' to 3')
1	CamR_*gmd-fcl* forward	TGTGACGGAAGATCACTTCG
2	CamR_*gmd-fcl* reverse	ACCAGCAATAGACATAAGCG
3	*gmd-fcl*_A	CGCGACTGTTCTCGACAATAAAGTCG
4	*gmd-fcl*_B	CACGACGATTTCCGGCAGTTTCTAC
5	*gmd-fcl*_C	GTAGAAACTGCCGGAAATCGTCGTG
6	*gmd-fcl*_D	GCTGGCAACCGATGTCTTTGTTGC
7	CamR_*waaF* forward	ATGGTGCCGTCCATTATTATCGCGGATGCCGGAAGTTA ACGAAGCTATTCACCAGCAATAGACATAAGCG
8	CamR_*waaF* forward	GATAACCCTCCGCAGCGTCACCTTTACGCACTTTGTGA TAGCCGGTAATCTGTGACGGAAGATCACTTCG
9	*waaF*_A	CAGGCAGATCTGACAAATCTGCGC
10	*waaF*_B	CACGACGATTTCCGGCAGTTTCTA C
11	*waaF*_C	GTAGAAACTGCCGGAAATCGTCGTG
12	*waaF*_D	CGTCATAGTTCTCTGCTTGTAGCGC

Figure S1 and Figure S2 demonstrate construction of the two knockout strains. Primers 3, 6, 9 and 12 anneal upstream and downstream of the *gmd-fcl* and *waaF* target genes, respectively. Together they yield a 2356 bp or 1686 bp PCR amplicon in unmodified *E. coli* K12 cells for *gmd-fcl* or *waaF* respectively. Upon insertion of the CamR cassette at the target locus, an approximately 1300 bp

(Δ*gmd-fcl*) or 1700 bp (Δ*waaF*) amplicon is produced. Primers 4, 5, 10 and 11 anneal to the CamR cassette, producing PCR products only when the CamR cassette has been inserted into the genome.

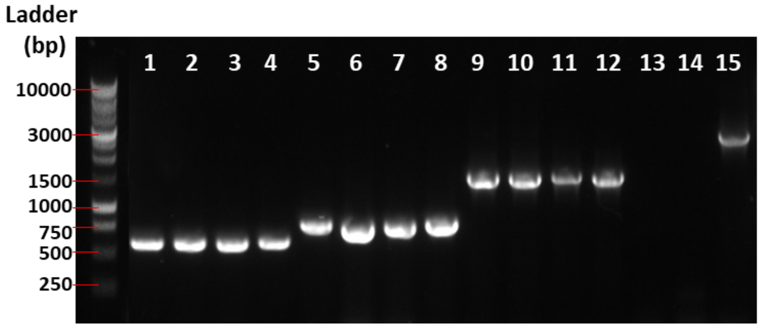

Figure S1. *Analysis of colony PCR to confirm generation of E. coli JM109(DE3)Δgmd-fcl. Lanes 1-4: primers A+B, clones 1-4; Lanes 5-8: primers C+D, clones 1-4; Lanes 8-12: primers A+D, clones 1-4; Lane 13: A+B, JM109(DE3) control; Lane 14: C+D JM109(DE3) control; Land 15: A+D JM109(DE3)_pSIM27 control. Theoretical PCR product lengths: A/B: 578 bp C/D: 728 bp A/D: 1279 bp A/D (Control): 2356 bp.*

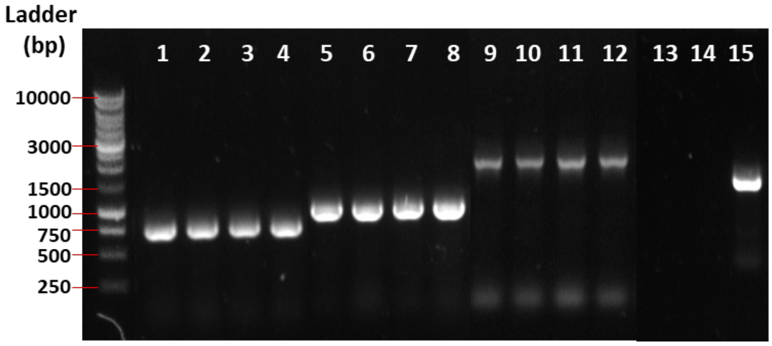

Figure S2. *Analysis of colony PCR to confirm generation of E. coli JM109ΔwaaF. Lanes 1-4: primers A+B, clones 1-4; Lanes 5-8: primers C+D, clones 1-4; Lanes 8-12: primers A+D, clones 1-4; Lane 13: A+B, control Lane 14: C+D control Land 15: A+D JM109_pSIM27 control. Theoretical PCR product lengths: A/B: 768 bp; C/D: 943 bp; A/D: 1686 bp; A/D (Control): 1684 bp.*

2.2 Strains and plasmids

Table S2. *Table of plasmids used in this study.*

**Name**	**Description**	**Reference**
pEdinbrick	Control plasmid: empty pSB1A2-derived vector with AmpR and pUC19-derived pMB1 origin of replication.	[2]
pRcsA	EPS overproduction plasmid: pSB1A2 backbone containing the *lac* promotor (part Bba J33207 [from Design of BioBricks, http://syntheticbiology.org/BioBricks/Part fabrication.html)] and *E. coli* K12 *rcsA* gene.	[3]
pFkp	Fkp expression plasmid: pRSFDuet1 backbone with *fkp* gene inserted into multiple cloning site 1 using NcoI and NotI restriction sites.	This study

	fkp (from *Bacteroides fragilis,* GenBank accession number: AY849806.1) gene sequence codon optimized for expression in *E. coli*:<br>ATGCAGAAACTGCTGAGCCTGCCGAGCAATCTGGTTCAGAGCTTTCATGAACT<br>GGAACGTGTTAATCGTACCGATTGGTTTTGTACCAGCGATCCGGTTGGTAAAA<br>AACTTGGTAGCGGTGGTGGCACCAGCTGGCTGCTGGAAGAATGTTATAATGA<br>ATACAGTGATGGTGCGACCTTTGGTGAATGGCTGGAAAAAGAAAAACGTATTC<br>TGCTGCATGCCGGTGGTCAGAGCCGTCGTCTGCCTGGTTATGCACCGAGCGGT<br>AAAATTCTGACACCGGTTCCGGTTTTTCGTTGGGAACGTGGTCAGCATCTGGG<br>TCAGAATCTGCTGTCACTGCAGCTGCCGCTGTATGAAAAAATCATGAGCCTGG<br>CACCGGATAAACTGCATACCCTGATTGCAAGCGGTGATGTTTATATTCGTAGT<br>GAAAAACCGCTGCAGAGCATTCCGGAAGCAGATGTTGTTTGTTATGGTCTGTG<br>GGTTGATCCGAGCCTGGCGACCCATCATGGTGTTTTTGCAAGCGATCGTAAAC<br>ATCCGGAACAGCTGGATTTTATGCTGCAGAAACCGAGTCTGGCAGAACTGGAA<br>AGCCTGAGCAAAACCCACCTGTTTCTGATGGATATTGGTATTTGGCTGCTGAG<br>CGATCGTGCAGTTGAAATTCTGATGAAACGTAGCCATAAAGAAAGCAGCGAA<br>GAACTGAAATATTACGATCTGTATAGCGATTTTGGTCTGGCACTGGGCACCCA<br>TCCGCGTATTGAAGATGAAGAAGTTAATACCCTGAGCGTTGCAATTCTGCCGC<br>TGCCTGGTGGCGAATTTTATCATTATGGTACAAGCAAAGAACTGATCAGCAGC<br>ACCCTGAGTGTTCAGAATAAAGTTTATGATCAGCGTCGCATCATGCACCGTAA<br>AGTTAAACCGAATCCGGCAATGTTTGTGCAGAATGCAGTTGTTCGTATTCCGCT<br>GTGTGCAGAAAATGCAGATCTGTGGATTGAAAACAGCCATATTGGTCCGAAAT<br>GGAAAATTGCAAGCCGTCATATTATCACCGGTGTTCCGGAAAATGATTGGAGC<br>CTGGCCGTTCCGGCAGGCGTTTGTGTTGATGTTGTTCCGATGGGTGATAAAGG<br>TTTTGTTGCACGTCCGTATGGTCTGGATGATGTTTTTAAAGGTGATCTGCGTGA<br>TAGCAAAACCACACTGACCGGTATTCCGTTTGGCGAATGGATGAGCAAACGTG<br>GTCTGAGCTATACCGATCTGAAAGGCCGTACCGATGATCTGCAGGCAGTTAGC<br>GTTTTTCCGATGGTTAATAGCGTTGAAGAACTGGGTTTAGTTCTGCGTTGGATG<br>CTGAGTGAACCGGAACTGGAAGAAGGTAAAAACATTTGGTTACGCAGCGAAC<br>ATTTTAGCGCAGATGAAATTAGTGCCGGTGCAAATCTGAAACGTCTGTATGCA<br>CAGCGTGAAGAATTTCGTAAAGGTAATTGGAAAGCACTGGCCGTGAATCATG<br>AAAAAAGCGTTTTTTATCAGCTGGATCTGGCAGATGCAGCCGAAGATTTTGTT<br>CGTTTAGGTCTGGATATGCCGGAACTGCTGCCGGAAGATGCACTGCAGATGA<br>GCCGTATTCATAATCGTATGCTGCGTGCCCGTATTCTGAAACTGGATGGTAAA<br>GATTATCGTCCGGAAGAACAGGCAGCATTTGATCTGCTGCGTGATGGTCTGCT<br>GGATGGCATTAGCAATCGTAAAAGCACCCCGAAACTGGACGTTTATAGCGATC<br>AGATTGTTTGGGGTCGTAGTCCGGTTCGTATTGATATGGCAGGCGGTTGGACC<br>GATACACCGCCTTATAGCCTGTATAGTGGTGGTAATGTTGTTAACCTGGCCATT<br>GAACTGAATGGTCAGCCTCCGCTGCAGGTTTATGTTAAACCGTGTAAAGATTTT<br>CATATCGTCCTGCGCTCAATCGATATGGGTGCAATGGAAATTGTTAGCACCTTT<br>GATGAACTGCAGGACTACAAAAAAATCGGTAGCCCGTTTAGTATTCCGAAAGC<br>AGCACTGTCACTGGCAGGTTTTGCCCCTGCATTTAGTGCAGTTAGCTATGCAAG<br>CCTGGAAGAACAACTGAAAGATTTTGGTGCAGGTATTGAAGTTACCCTGCTGG<br>CAGCAATTCCTGCAGGTAGCGGTCTGGGCACCAGTAGCATTCTGGCAAGCACC<br>GTTCTGGGTGCCATTAATGATTTTTGTGGTCTGGCGTGGGATAAAAACGAAAT<br>TTGTCAGCGTACCCTGGTTCTGGAACAGTTACTGACCACAGGTGGTGGTTGGC<br>AGGATCAGTATGGTGGTGTTCTGCAGGGTGTTAAACTGCTGCAGACCGAAGC<br>CGGTTTTGCACAGAGTCCGCTGGTTCGTTGGCTGCCGGATCACCTGTTTACCCA<br>TCCGGAATATAAAGATTGTCATCTGCTGTATTATACCGGCATTACCCGTACCGC<br>AAAAGGTATTCTGGCCGAAATTGTGAGCAGCATGTTTCTGAATAGCAGCCTGC<br>ATCTGAACCTGCTGTCAGAAATGAAAGCACATGCACTGGATATGAATGAAGCA<br>ATTCAGCGTGGTAGCTTTGTTGAATTTGGTCGTCTGGTGGGTAAAACCTGGGA	

	ACAGAACAAAGCCCTGGATAGCGGCACCAATCCTCCGGCAGTTGAAGCCATTA TTGATCTGATCAAAGATTATACCCTGGGCTATAAACTGCCAGGTGCCGGTGGC GGTGGTTATCTGTATATGGTTGCAAAAGATCCGCAGGCAGCAGTGCGTATTCG TAAAATCCTGACCGAAAATGCTCCGAATCCGCGTGCACGTTTTGTTGAAATGA CCCTGTCAGATAAAGGCTTTCAGGTTAGCCGTAGCTAA	

3 Supplementary quantification methods

3.1 Colanic acid quantification using plate reader

The colanic acid quantification assay was successfully miniaturized for high throughput analysis using a BMGLabtech FluoSTAR Omega plate reader. Purified EPS samples were diluted in Milli-Q water depending on their predicted concentration to fit within the standard curve. 111 µL of the diluted EPS mixture was added to 500 µL H_2SO_4/water (6:1 v/v) and the mixture was heated to 95 °C for 30 minutes then cooled to room temperature. For each sample, to a well of a flat-bottomed 96-well place was added (a) 5 µL cysteine hydrochloride (Cys·HCl, 3% stock solution) or (b) 5 µL Milli-Q water. To each of these was added 200 µL of the cooled acidified EPS mixture. The absorbance of both (a) and (b) was measured at 396 and 427 nm. The absorbance measurements at both 396 and 427 nm without Cys·HCl were subtracted from those with Cys·HCl to provide background corrected A_{396} and A_{427} values. The final absorbance values were calculated by subtracting A_{427} from A_{396}. The result was directly correlated to fucose concentration by using a fucose standard curve ranging from 5 µg/mL to 100 µg/mL. A separate standard curve was prepared in parallel to the samples for each analytical run to account for differences in incubation times. A representative standard curve is shown in Figure S3.

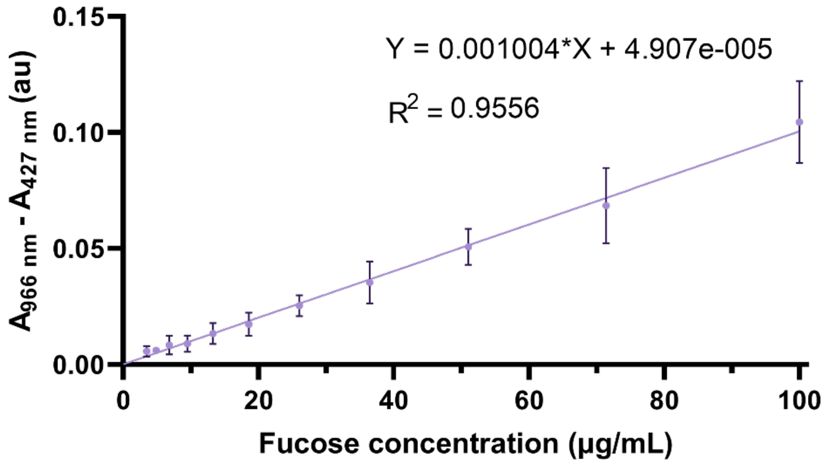

Figure S3. *Representative standard curve for the quantification of fucose concentration.*

3.2 Quantitative analysis of total carbohydrate content

The total carbohydrate content of the samples was quantified by the anthrone-sulfuric acid assay based on a protocol from Rondel and coworkers[5]. The purified EPS samples were diluted in Milli-Q water depending on their expected concentration to fit within the standard curve, and 400 µL aliquots were added to a glass vial for each sample. To this was added 800 µL of a freshly prepared anthrone solution (2% w/v in 96% aq. H_2SO_4). The mixture was heated at 60 °C for 30 minutes then cooled to room temperature. The absorbance of the resulting solution at 620 nm was measured and correlated to glucose concentration using a standard curve ranging from 5 µg/mL to 100 µg/mL. A representative standard curve is shown in Figure S4.

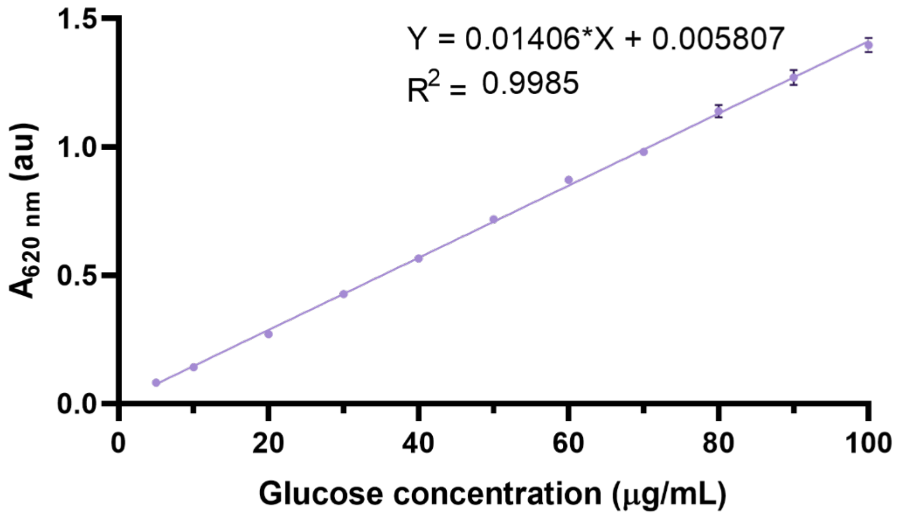

Figure S4. *Representative standard curve for the quantification of glucose concentration.*

4 EPS production supplementary methods

4.1 Optimization of colanic acid production

Glucose concentration

E. coli JM109_pRcsA was grown in minimal M9 or 1xMDM media supplemented with glucose at concentrations of 5% (w/v) or 0.5% (w/v). All cultures were incubated at 37 °C with rotary shaking at 220 rpm 24 hours post-induction.

Nitrogen source

M9 and 1xMDM media were prepared according to the recipes in Section 1.2, but excluding the nitrogen source. Ammonium chloride or ammonium sulphate were added at a concentration of 1 g/L and proline was added either at a concentration of 1 g/L or 5.4 g/L. All cultures were incubated at 37 °C and 220 rpm for 24 hours post-induction.

Temperature and addition of trace metals

M9 with either NH_4Cl (1 g/L) or proline (5.4 g/L) was supplemented with $FeSO_4 \cdot 7H_2O$ and $CuSO_4 \cdot 5H_2O$, both to 1 mg/L final concentration to test their effect on the growth and production of exopolysaccharides by *E. coli* JM109_pRcsA. The cultures were incubated either at 37 °C or 19 °C and 220 rpm until they reached an OD_{600} = 0.6 at which point they were induced with isopropyl β-D-1-thiogalactopyranoside (IPTG) (0.5 mM final concentration). Further incubation took place under the same conditions over 24 hours, except the cultures previously grown at 37 °C were either maintained at the same temperature or incubated at 19 °C.

Time

Time course experiments were carried out in M9 minimal media supplemented with proline (5.4 g/L) as the nitrogen source, trace metals $FeSO_4 \cdot 7H_2O$ and $CuSO_4 \cdot 5H_2O$ at a final concentration of 0.001 g/L and 5 % w/v glucose as the carbon source. All cultures were incubated at 19 °C and 220 rpm for approximately 24 hours until the induction point. Further incubation was carried out under the same conditions for 65, 90 or 115 hours. Growth curves were obtained by taking 200 µL aliquots at different time points and measuring the OD_{600}.

4.2 Procedure for preparation of fluorescence microscopy samples

Cultures of JM109(DE3)Δ*gmd-fcl*_pRcsA_pFkp were grown as described with Fuc-N_3 to produce azide-labelled colanic acid, or with fucose to produce non-labelled colanic acid as the control culture. Both cultures were treated identically throughout. Fluorescent labelling was performed as described, and the finished reactions dialyzed for 50 hours in water in 3.5 kDa molecular weight cut-off dialysis cassettes, changing the dialysis water every 8-16 hours.

For fluorescence microscopy, the dialyzed cultures were diluted with water to OD_{600} = 0.2 and 100 µL was transferred onto an air-dried glass slide coated with 0.05 % (w/v) poly-D-lysine and allowed to bind for 30 minutes before washing with water. The slide was air-dried and 50 µL ProLong™ Diamond Antifade (Thermo Fisher Scientific) was dropped onto the sample before covering with a coverslip. Images were taken using a Nikon Eclipse Ti2 microscope (Nikon Europe, Amstelveen, Netherlands) equipped with a x100 objective and Prime 95B sCMOS camera (Teledyne Photometrics, Birmingham UK). Fluorescence was detected using a 488 nm excitation filter, Sedat Quad dichroic and 520 nm emission filter (Chroma Technology Corp, Bellows Falls). All images were analyzed post acquisition using Fiji[6].

5 Synthesis

1,2,3,4-di-*O*-isopropylidene-α-L-galactopyranose

L-Galactose (245 mg, 1.35 mmol) was suspended in acetone (5 mL) and $ZnCl_2$ (370 mg, 2.7 mmol) was added followed by conc. H_2SO_4 (25 µL, 0.46 mmol) and the solution stirred at room temperature for 24 hours. To the orange solution was added $NaHCO_3$ (5 mL; sat. aq.) and the solution was stirred for 20 minutes, during which time it turned colorless. The solution was filtered through celite and the filtrate extracted with ether (3 × 20 mL). The organic phases were combined, dried over $MgSO_4$ and concentrated under reduced pressure to yield the product as a yellow oil which was used without further purification (288 mg, 82% yield); $\mathbf{R_f}$ (3:1 Hexanes/EtOAc) = 0.3; **^{1}H NMR** (600 MHZ, $CDCl_3$) δ 5.59 (1H, d, *J* = 5.0 Hz, *H*-1), 4.64 (1H, dd, *J* = 7.9, 2.4 Hz, *H*-3), 4.36 (1H, dd, *J* = 5.0, 2.4 Hz, *H*-2), 4.30 (1H, dd, *J* = 7.9, 1.6 Hz, *H*-4), 3.93 – 3.84 (2H, m, *H*-5, H-6_α), 3.77 (1H, ddd, *J* = 12.5, 6.8, 2.8 Hz, H-6_β), 1.56 (3H, s, CH_3), 1.48 (3H, s, CH_3), 1.36 (6H, br s, 2 × CH_3); **^{13}C NMR** (126 MHz, $CDCl_3$) δ 109.4 (C), 108.6 (C), 96.3 (CH), 71.6 (CH), 70.8 (CH), 70.6 (CH), 68.0 (CH), 62.3 (CH_2), 26.0 (CH_3), 25.9 (CH_3), 24.9 (CH_3), 24.3 (CH_3). Spectroscopic data matches the literature[7].

6-azido-1,2,3,4-di-*O*-isopropylidene-6-deoxy-α-L-galactopyranoside

To 1,2,3,4-di-*O*-isopropylidene-α-L-galactopyranose (288 mg, 1.1 mmol) was added anhydrous DCM (5 mL) and *N*,*N*-diisopropylethylamine (1.3 mL, 7.5 mmol) under argon and the solution cooled on ice. Tf_2O (0.5 mL, 3.0 mmol) was added to the solution dropwise over 5 minutes and the solution stirred for a further 15 minutes. TLC (3:1 Hexanes/EtOAc, visualized using permanganate) showed product (R_f = 0.8) and remaining starting material (R_f = 0.3). A further portion of Tf_2O (0.25 mL, 1.5 mmol) was added dropwise to the solution, whereupon TLC confirmed complete consumption of starting material. Cold water (20 mL) was added to the solution which was extracted with DCM (3 × 20 mL). The organic phases were combined, dried over Na_2SO_4 and concentrated under reduced pressure to yield crude 1,2:3,4-di-*O*-isopropylidene-6-*O*-(trifluoromethanesulfonyl)-α-L-galactopyranose as a red oil. The oil was dissolved in DMSO (5 mL), NaN_3 (0.7 g, 10.8 mmol) was added and the solution stirred at room temperature for 20 hours. Water (30 mL) was added and the solution was extracted with Et_2O (3 × 20 mL). The combined organic phases were dried over $MgSO_4$ and concentrated under reduced pressure. Purification by flash chromatography using 9:1 Hexanes/EtOAc gave the product as a colorless oil (200 mg, 64% yield); $\mathbf{R_f}$ (9:1 Hexanes/EtOAc) = 0.2; **^{1}H NMR** (500 MHz, $CDCl_3$) δ 5.57 (1H, d, *J* = 5.0 Hz, *H*-1), 4.65 (1H, dd, *J* = 7.9, 2.5 Hz, *H*-3), 4.36 (1H, dd, *J* = 5.0, 2.5 Hz, *H*-2), 4.22 (1H, dd, *J* = 7.9, 2.0 Hz, *H*-4), 3.94 (1H, ddd, *J* = 7.5, 5.3, 2.0 Hz, *H*-5), 3.53 (1H, dd, *J* = 12.7, 7.9 Hz, H-6_a), 3.39 (1H, dd, *J* = 12.7, 5.3 Hz, H-6_b), 1.57 (3H, s, CH_3), 1.48 (3H, s, CH_3), 1.37 (3H, s. CH_3), 1.36 (3H, s, CH_3); **^{13}C NMR** (126 MHz, $CDCl_3$) δ 109.6 (C), 108.8 (C), 96.4 (CH), 71.2 (CH), 70.8 (CH), 70.4 (CH), 67.0 (CH), 50.7 (CH_2), 26.0 (CH_3), 26.0 (CH_3), 24.9 (CH_3), 24.44 (CH_3). Spectroscopic data matches the literature[8].

6-Azido-L-fucose

6-azido-1,2,3,4-di-*O*-isopropylidene-6-deoxy-α-L-galactopyranoside (200 mg, 0.7 mmol) was dissolved in TFA (5 mL; 80% aq.) and stirred at room temperature for 3 hours. The solution was concentrated under reduced pressure yielding a yellow oil. The product was obtained by recrystallisation from EtOH (1 mL) as a white solid. The recrystallisation liquor was concentrated to approximately 200 µL and further product was isolated by layer diffusion crystallization with ether (84 mg, 58% yield); $\mathbf{R_f}$ (15% MeOH in DCM) = 0.2; $\boldsymbol{\alpha_D}$ = -66.4 ° (*c* = 0.8, H_2O), [Lit[9] = -56.4 °, (*c* = 1, H_2O)]; ^{1}**H NMR** (600 MHz, D_2O) δ 5.30 (1H, dd, *J* = 8.2, 3.8 Hz, α*H*-1), 4.64 (1H, d, *J*=7.9 Hz, β*H*-1), 4.23 (1H, ddd, *J* = 8.9, 4.4, 1.1 Hz, α*H*-5), 3.99 (1H, dd, *J* = 3.3, 1.2 Hz, α*H*-4), 3.93 (1H, dd, *J* = 3.4, 1.1 Hz, β*H*-4), 3.89 (1H, dd, *J* = 10.3, 3.4 Hz), 3.85 (1H, ddd, *J*=8.6, 4.4, 1.1 Hz, β*H*-5), 3.82 (1H, dd, *J* = 10.3, 3.6 Hz, α*H*-2), 3.68 (1H, dd, *J* = 9.9, 3.5 Hz, β*H*-3), 3.62 (1H, dd, *J*=13.0, 8.5 Hz, β*H*-6_a), 3.57 (1H, dd, *J* = 13.0, 8.6 Hz, α*H*-6_a), 3.54 – 3.48 (3H, m, β*H*-2, α*H*-6_b + β*H*-6_b); ^{13}**C NMR** (126 MHz, D_2O) δ 96.4 (CH-β), 92.4 (CH-α), 73.4 (CH-β), 72.66 (CH-β), 71.7 (CH-β), 69.6 (CH-α), 69.1 (CH-β), 69.0 (CH-α), 68.9 (CH-α), 68.2 (CH-α), 50.9 (CH-α), 50.7 (CH-β); **HRMS** (ESI+) $[M+H]^+$ found 206.0782, $C_6H_{12}N_3O_5$ requires 206.0771. Spectroscopic data matches the literature[10].

6 Supplementary data

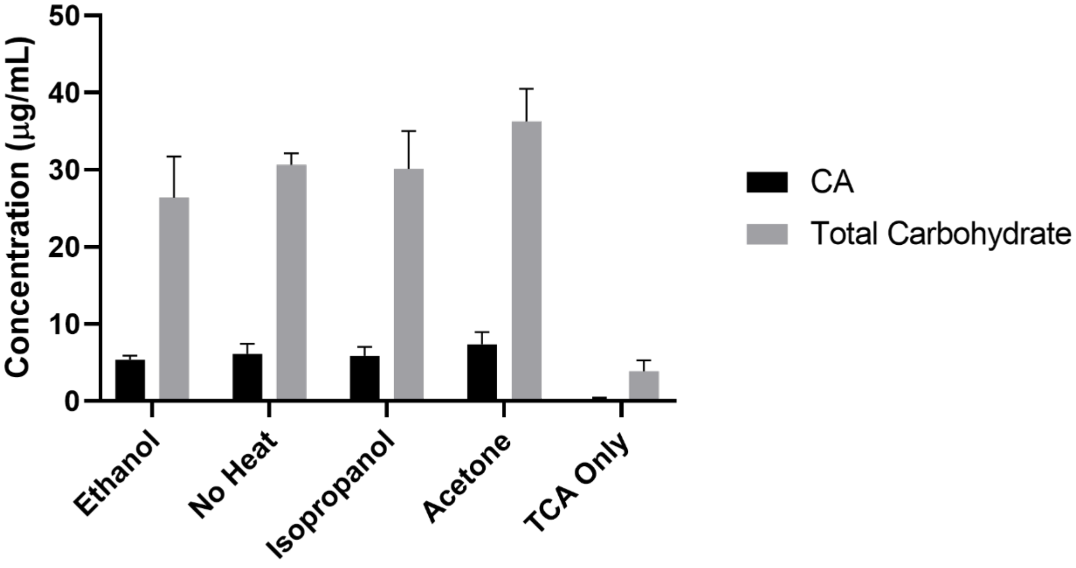

Figure S5. *Effect of the extraction procedure and solvent used in the CA production (black) and the total carbohydrate content (grey). Error bars represent the standard deviation of values from three independent biological replicates. TCA: trichloroacetic acid.*

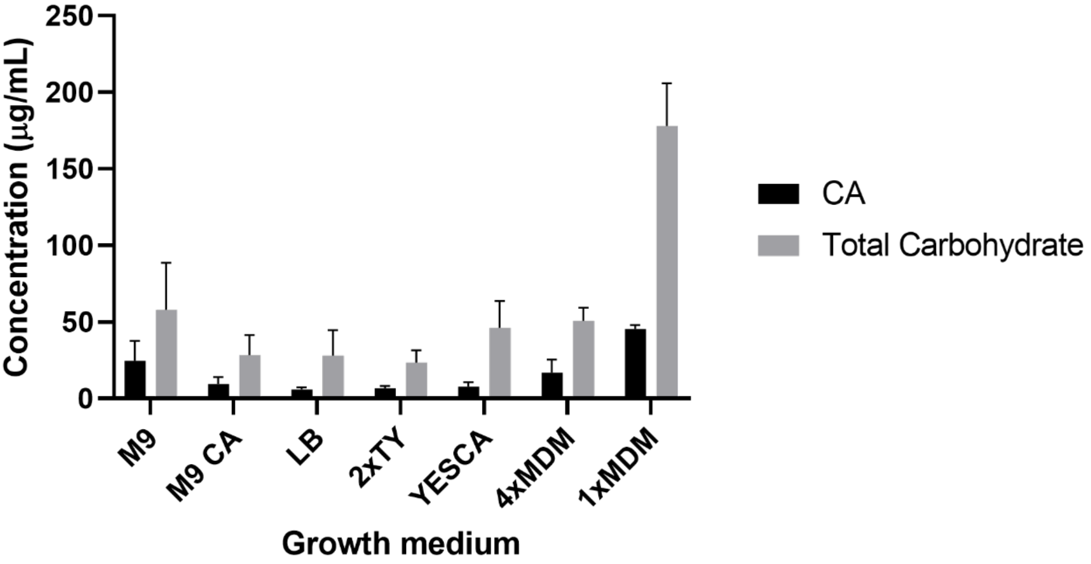

Figure S6. *Effect of the media used for the growth of E. coli JM109_pRcsA in the production of CA (black) and total carbohydrate content (grey). Error bars represent the standard deviation of values from three independent biological replicates.*

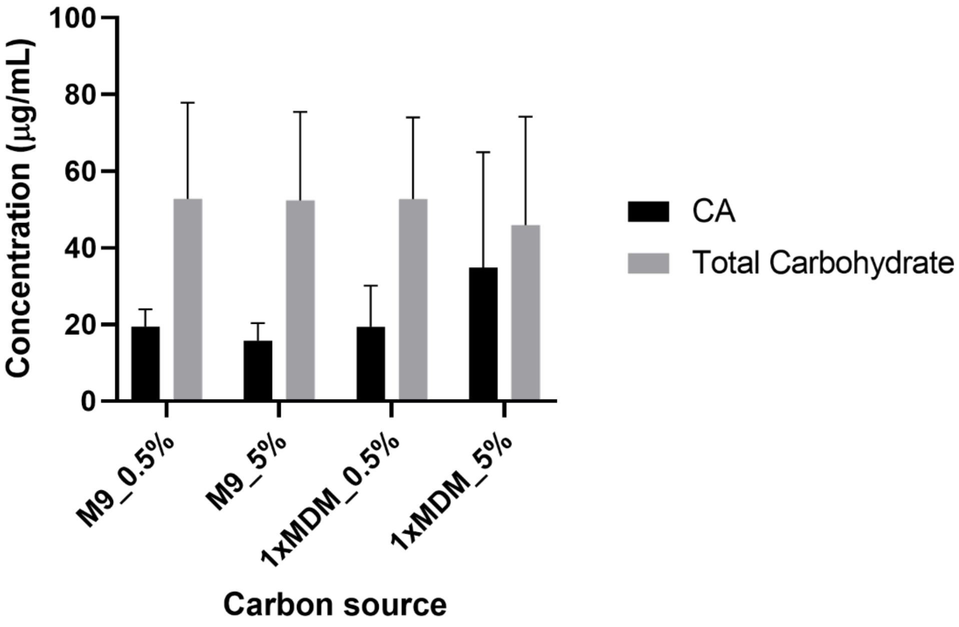

Figure S7. *Effect of the glucose percentage used either in M9 or 1xMDM (0.5% or 5%) in the production of CA (black) and total carbohydrate production (grey). Error bars represent the standard deviation of values from three independent biological replicates.*

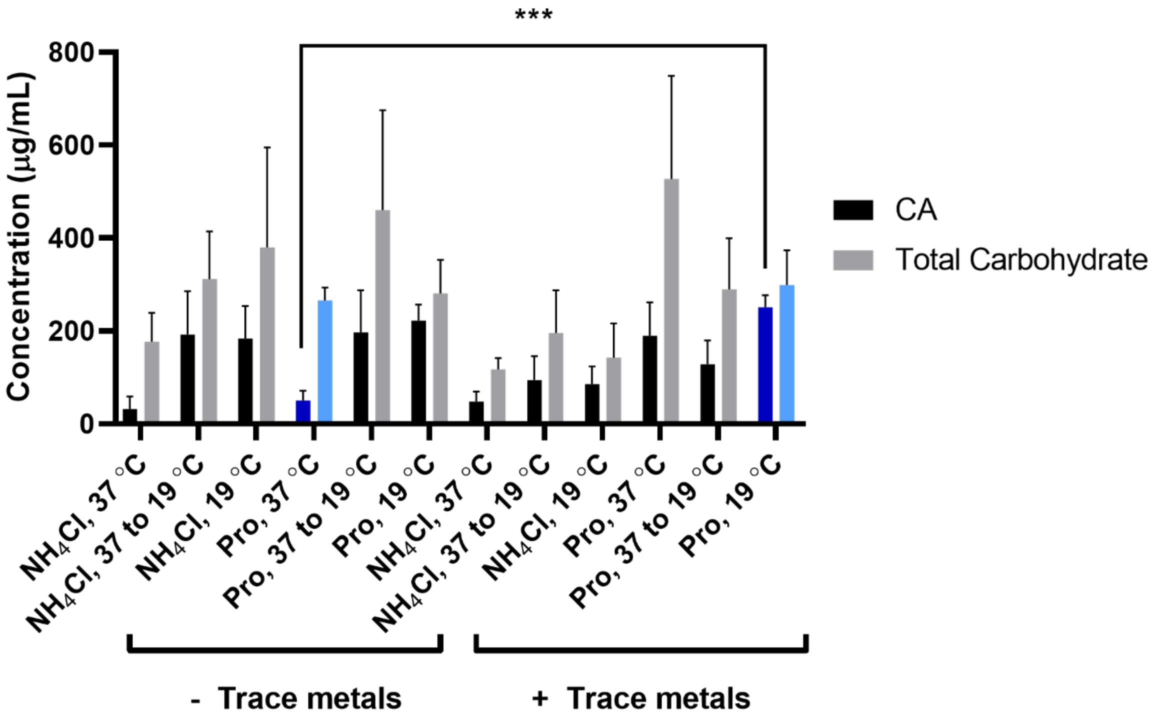

Figure S8. *Effect of the incubation temperature and the addition of Cu(II) and Fe(II) as trace metal ions in the production of CA (black) and the total carbohydrate content (grey). Data from the proline control cultures and proline cultures incubated at 19 °C with the addition of metal ions are highlighted in blue. Error bars represent the standard deviation of values from three independent biological replicates; ***P<0.0001 (Dunnet's test).*

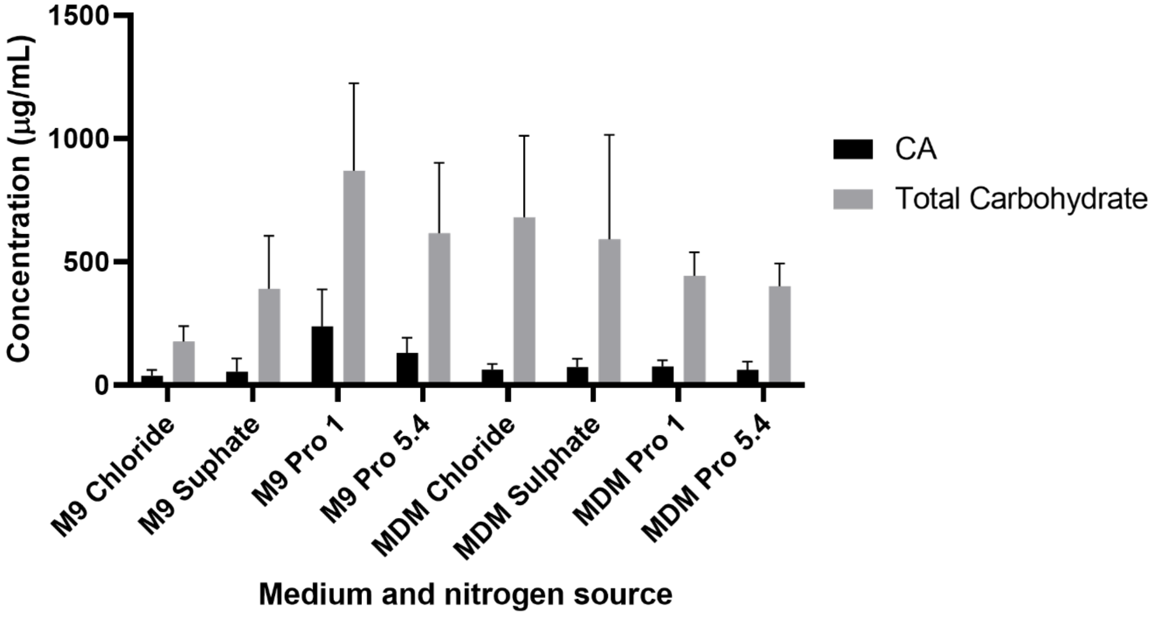

Figure S9. *Effect of the nitrogen source used in the growth media in the production of CA (black) and the total carbohydrate content (grey). Error bars represent the standard deviation of values from three independent biological replicates.*

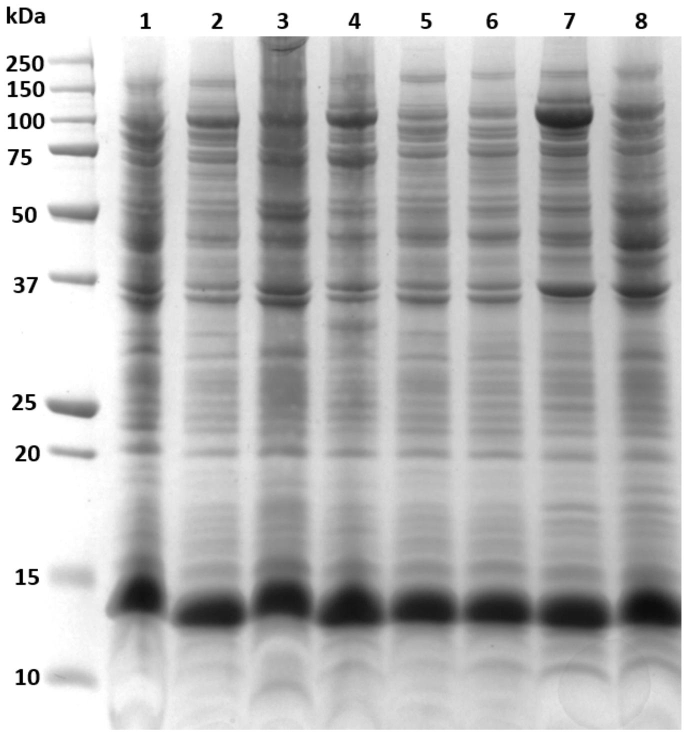

Lane	Strain	Pre-induction temp. (°C)	Media
**1**	JM109(DE3)Δ*gmd-fcl*_pRcsA_pFkp	22	M9-EPS
**2**	JM109(DE3)Δ*gmd-fcl*_pRcsA_pFkp	37	M9-EPS
**3**	JM109(DE3)Δ*gmd-fcl*_pRcsA_pFkp	22	LB
**4**	JM109(DE3)Δ*gmd-fcl*_pRcsA_pFkp	37	LB
**5**	JM109(DE3)Δ*gmd-fcl*_pRsfDuet1	37	M9-EPS
**6**	JM109(DE3)Δ*gmd-fcl*_pRsfDuet1	37	LB
**7**	BL21(DE3)_pFkp	37	LB
**8**	BL21(DE3)_pFkp	22	LB

Figure S10. *SDS-PAGE analysis of Fkp expression. All cultures were incubated at 19 °C for 24 hours post-induction with IPTG. JM109(DE3)Δgmd-fcl_pRsfDuet1 was included as a negative control for each condition; BL21(DE3)_pFkp was included as a positive control for each condition. Theoretical size of Fkp: 106 kDa.*

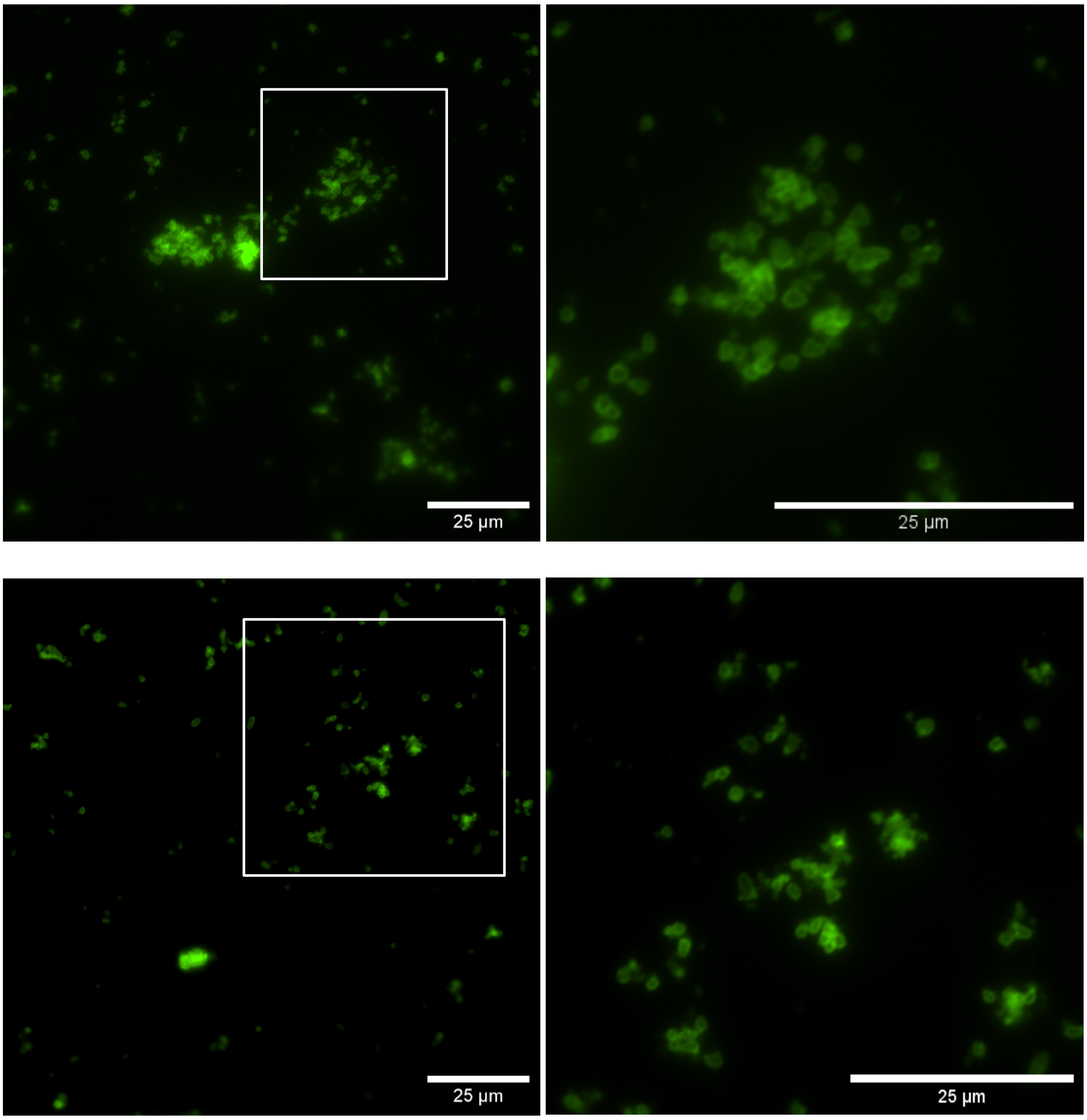

Figure S11. *Fluorescence microscopy images of fluorescently labelled colanic acid attached to E. coli (JM109(DE3)Δgmd-fcl_pRcsA_pFkp) cells. Two regions of the same slide are shown (left hand side, top and bottom), each with a white box showing the zoomed region displayed on the right.*

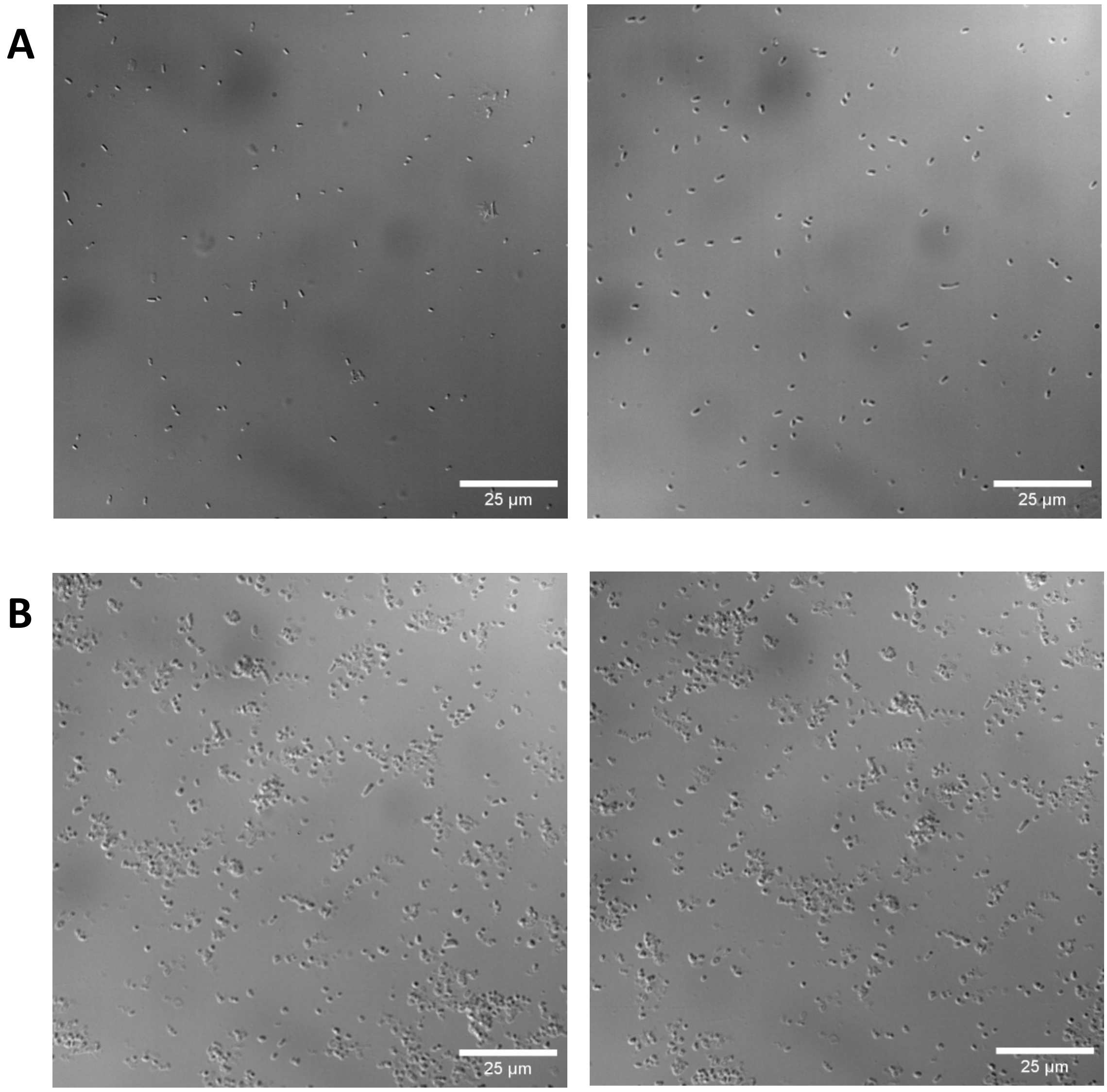

Figure S12. *Brightfield images of E. coli JM109(DE3)Δgmd-fcl_pRcsA_pFkp cells subjected to click reaction conditions. A: Cells grown in the presence of fucose (control, fluorescence not observed by microscopy); B: cells grown in the presence of Fuc-N_3 (fluorescence observed by microscopy as shown in Figure 11). Two images of the same slide are shown in each case.*

7 References

1 J. A. Sawitzke, L. C. Thomason, N. Costantino, M. Bubunenko, S. Datta and D. L. Court, *Methods Enzymol.*, 2007, **421**, 171–199.

2 Catalog - parts.igem.org, http://parts.igem.org/Catalog, (accessed 15 December 2021).

3 N. Joshi, B. T. Ngwenya and C. E. French, Enhanced resistance to nanoparticle toxicity is conferred by overproduction of extracellular polymeric substances, *J. Hazard. Mater.*, 2012, **241**–**242**, 363–370.

4 B. Obadia, S. Lacour, P. Doublet, H. Baubichon-Cortay, A. J. Cozzone and C. Grangeasse, Influence of tyrosine-kinase Wzc activity on colanic acid production in *Escherichia coli* K12 cells, *J. Mol. Biol.*, 2007, **367**, 42–53.

5 C. Rondel, C. E. Marcato-Romain and E. Girbal-Neuhauser, Development and validation of a colorimetric assay for simultaneous quantification of neutral and uronic sugars, *Water Res.*, 2013, **47**, 2901–2908.

6 J. Schindelin, I. Arganda-Carreras, E. Frise, V. Kaynig, M. Longair, T. Pietzsch, S. Preibisch, C. Rueden, S. Saalfeld, B. Schmid, J. Y. Tinevez, D. J. White, V. Hartenstein, K. Eliceiri, P. Tomancak and A. Cardona, Fiji: An open-source platform for biological-image analysis, *Nat. Methods*, 2012, **9**, 676–682.

7 J. Chan, A. Lu and A. J. Bennet, Turnover Is rate-limited by deglycosylation for micromonospora viridifaciens sialidase-catalyzed hydrolyses: Conformational implications for the Michaelis complex, *J. Am. Chem. Soc.*, 2011, **133**, 2989–2997.

8 Ö. Işılar, A. Bulut, A. Sahin Yaglioglu, İ. Demirtaş, E. Arat and M. Türk, Synthesis and biological evaluation of novel urea, thiourea and squaramide diastereomers possessing sugar backbone, *Carbohydr. Res.*, 2020, **492**, 107991.

9 J. A. May and A. C. Sartorelli, Synthesis and biological activity of potential antimetabolites of L-fucose, *Potential Antimetab. h-Fucose J. Med. Chem.*, 1979, **22**, 971.

10 C.-H. Wong, R. Alajarin, F. Morís-Varas, O. Blanco, E. Garcia-Junceda and G. Tetrahedron Lett, Enzymatic synthesis of L-fucose and analogs, *J. Org. Chem.*, 1995, **60**, 7360–7363.
